# Single gene locus changes perturb complex microbial communities as much as apex predator loss

**DOI:** 10.1038/ncomms9235

**Published:** 2015-09-10

**Authors:** Deirdre McClean, Luke McNally, Letal I. Salzberg, Kevin M. Devine, Sam P. Brown, Ian Donohue

**Affiliations:** 1 Department of Zoology, School of Natural Sciences, Trinity College Dublin, Dublin D2, Ireland; 2 Trinity Centre for Biodiversity Research, Trinity College Dublin, Dublin D2, Ireland; 3 Centre for Immunity, Infection and Evolution, School of Biological Sciences, University of Edinburgh, Edinburgh EH9 3FL, UK; 4 Institute of Evolutionary Biology, School of Biological Sciences, University of Edinburgh, Edinburgh EH9 3FL, UK; 5 Smurfit Institute of Genetics, School of Genetics and Microbiology, Trinity College Dublin, Dublin D2, Ireland; 6 School of Biology, Georgia Institute of Technology, Atlanta, Georgia 30332, USA

## Abstract

Many bacterial species are highly social, adaptively shaping their local environment through the production of secreted molecules. This can, in turn, alter interaction strengths among species and modify community composition. However, the relative importance of such behaviours in determining the structure of complex communities is unknown. Here we show that single-locus changes affecting biofilm formation phenotypes in *Bacillus subtilis* modify community structure to the same extent as loss of an apex predator and even to a greater extent than loss of *B. subtilis* itself. These results, from experimentally manipulated multitrophic microcosm assemblages, demonstrate that bacterial social traits are key modulators of the structure of their communities. Moreover, they show that intraspecific genetic variability can be as important as strong trophic interactions in determining community dynamics. Microevolution may therefore be as important as species extinctions in shaping the response of microbial communities to environmental change.

## Abstract (web summary)

Some species of social bacteria can chemically modify their nutrient environments, which may influence community interactions. Here, McClean *et al.* show that changes at a single gene locus in a biofilm-forming bacteria can perturb community structure to the same extent as the loss of an apex predator.

## Graphical abstract

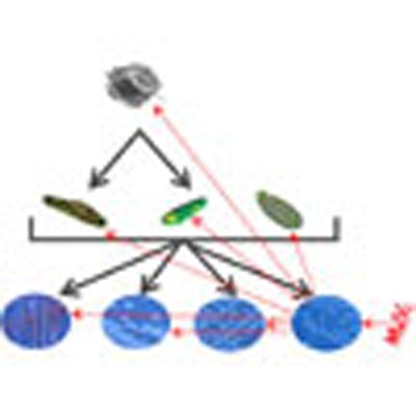

Understanding the drivers of the composition and dynamics of biotic communities is one of the central goals of ecology. Most research in the field has examined how the extinction, invasion or shifts in the abundance of species affect the composition and stability of the remainder of the community^1^,^2^,^3^,^4^,^5^. However, variation of traits within a species could also be a major factor driving the dynamics of community composition^6^,^7^,^8^,^9^,^10^. In particular, ‘niche construction' traits^11^, by which organisms modify their local biotic and abiotic environments through their actions and secretions, may be of great importance in determining community dynamics.

Bacteria display an extensive array of social behaviours through which they can modify their local environments, such as secretions of toxins that kill competitors and exoenzymes that modify their nutrient environments^12^,^13^,^14^. The secretion of polymeric matrices to form densely populated and highly structured biofilm is a striking example of such a niche construction trait^15^. These complex biofilms create microniches within their walls but can also alter their external environment extensively by, for example, depleting oxygen^16^ and modifying fluid dynamics^17^. Recent experiments using highly simplified two-species communities suggest that changes in biofilm formation phenotypes could affect interactions among species and, by extension, community composition^18^,^19^,^20^. However, whether these traits have any effect in more complex multitrophic communities, and their magnitude relative to important biotic perturbations such as species extinctions, remains unknown.

To assess the effects of variation in bacterial social traits on the structure of multitrophic microbial communities, we used experimental freshwater microcosm communities consisting of four bacterial species (*Bacillus subtilis*, *Aeromonas* sp.*, Klebsiella* sp. and *Serratia marcescens*), three protist primary consumers (*Paramecium caudatum*, *P. aurelia* and *Colpidium* sp.) and one apex specialist predator (*Didinium nasutum*; Fig. 1a). We manipulated the biofilm formation phenotype of *B. subtilis* using two separate deletions of the regulatory genes *sinI* and *sinR*. The *sinR* gene is a master regulator of biofilm formation in *B. subtilis*^21^. The Δ*sinI* mutant is continually motile and does not form cell chains (reduced, or constitutive off for, biofilm formation). The Δ*sinR* mutant grows as non-motile chains (hyper, or constitutive on for, biofilm formation), while the wild-type (WT) strain 3610 is plastic for this trait and shows an intermediate level of both biofilm formation and motility (Fig. 1b). As an additional procedural control, we also used a mutant *phoA* deletion of *B. subtilis*, which is unable to produce alkaline phosphatase in response to phosphate starvation^22^. As this latter mutation has no ‘niche construction' effects, it would be expected to have little effect on community structure in our experimental system.

To benchmark the ability of the different biofilm formation phenotypes to influence community structure, we assessed the impact of perturbing biofilm traits relative to the loss of the apex predator from the system (that is, *Didinium*; Fig. 1). The extinction of species, especially those belonging to upper trophic levels, can have particularly significant consequences for ecosystem structure and functioning^3^,^23^,^24^. Predators tend to be the key drivers of community dynamics and, consequently, predator loss is considered as one of the most profound biotic perturbations that can occur^3^,^25^,^26^. Furthermore, besides simulating experimentally the extinction of the apex predator in our system, we also established a treatment without *B. subtilis* to examine the relative importance of variation in biofilm-forming phenotypes compared with the complete absence of the species.

We found that the presence of the different *B. subtilis* mutants perturbed the structure of the mesocosm communities as much as the loss of the apex predator *Didinium* and even to a greater extent than loss of *B. subtilis* itself. These results indicate remarkable scope for fine-scale genetic variability within populations of a single species to regulate the structure, dynamics and functioning of whole communities.

## Results

### Community composition

The structure of the microcosm communities varied significantly among our experimental treatments (multivariate analysis of variance (MANOVA), Pillai's Trace=0.762, *F*_4,45_=1.56, *P=*0.036; Fig. 2). As expected, removal of the predatory *Didinium* initiated a strong trophic cascade, increasing the densities of some primary consumers significantly (particularly *P. caudatum* and *Colpidium*) and causing a consequent drop in the densities of some bacteria (most notably *Aeromonas*; Fig. 2a,d). Microcosms with suppressed biofilm production (that is those containing Δ*sinI*; Fig. 2e) contained increased densities of the primary consumer *P. caudatum* and, to a lesser extent, *Didinium*, while all other species were present in reduced densities. In contrast, even though microcosms with enhanced biofilm production (that is, those containing Δ*sinR*; Fig. 2f) also contained increased densities of *P. caudatum*, densities of *Didinium* were relatively low, while those of the biofilm-producing bacterium *B. subtilis* increased considerably.

### Magnitude of mutant effects

To assess the relative magnitude of the effects of our experimental manipulations, we tested whether they diverged in structure from the control treatment more than expected by chance (Fig. 3). We found that neither the removal of *B. subtilis* nor the replacement of *B. subtilis* WT with the Δ*phoA* metabolic mutant significantly altered the structure of the microcosm communities from that of the control, whereas removal of the top predator *Didinium* generated significant changes in overall community structure (Fig. 3b).

Compared with these benchmarks, we found that both biofilm mutant treatments (that is, those containing Δ*sinI* or Δ*sinR*) generated significant changes in community structure, and that these changes were comparable in magnitude to changes that occurred after removal of the top predator (two-tailed *P* values calculated by bootstrapping: *Didinium* removed versus *ΔsinR*=0.91, *Didinium* removed versus *ΔsinI*=0.78, *ΔsinR* versus *ΔsinI*=0.83; Fig. 3b). Moreover, replacement of WT *B. subtilis* by either of the social mutants used in the experiment had a greater effect on community structure than the complete removal of the species (Fig. 3b). The responses of species other than *B. subtilis* along the observed gradient of biofilm produced were, however, non-monotonic (Fig. 4 and Supplementary Table 1). The effects of these mutations on overall community structure were, therefore, driven indirectly by changes in the interactions among species rather than being simply a direct consequence of changes in the abundance of *B. subtilis*. Subsequent experiments examining the individual effects of each of the three primary consumer species used in the experiment revealed that, while each consumer species had similar effects on bacterial community structure across all *B. subtilis* treatments (MANOVA, Pillai's Trace=1.05, *F*_16,72_=1.6, *P=*0.09), bacterial communities, nonetheless, varied significantly among microcosms containing the different *B. subtilis* phenotypes (Pillai's Trace=0.93, *F*_8,32_=3.5, *P=*0.005; Supplementary Fig. 1). *Post hoc* tests showed that bacterial community structure was similar in microcosms containing WT and *ΔsinR* but that these systems differed significantly (*P*<0.01 in every case) from microcosms containing *ΔsinI.* Together, these results suggest that different mechanisms underpinned the shifts in overall community structure caused by the presence of the different social mutants in our main experiment. While the reduction of biofilm in microcosms containing *ΔsinI* likely altered interactions among bacterial species which, in turn, altered overall community structure (a ‘bottom-up' effect), differences between the microcosms containing *B. subtilis* WT and *ΔsinR* appear to be a consequence of the presence of multiple species of consumers triggering complex shifts in interactions among species (a ‘top-down' effect), and were not driven directly by changes in the interactions of a single species.

## Discussion

Our results show that single-locus changes in genes regulating species' niche construction and motility traits can modify communities to a greater extent than loss of the niche-constructing species itself and to the same extent as the loss of an apex predator. These findings have novel and important implications for our understanding of community dynamics, demonstrating that intraspecific genetic variability at the scale of even a single gene locus can be as important as strong trophic interactions in determining the structure of complex communities. While it is becoming increasingly apparent that intraspecific trait variation can moderate community dynamics (for example, refs 7, 9, 10, 27), our findings indicate remarkable scope for fine-scale genetic variability within populations to regulate the structure, dynamics and functioning of whole ecosystems.

Recent studies suggest that shifts in the composition of the human microbiota are of great importance for human disease^28^,^29^,^30^. These compositional shifts are often caused by changing conditions within the host or invasions into these communities^28^,^30^. However, our results suggest that changes in social traits within resident species could be as important as these other known factors in shaping the composition of host microbiota and other communities. We found that communities in which the behaviour of a single species was constrained to constitutive phenotypes differed remarkably in terms of their structure and dynamics from those containing plastic phenotypes that could switch between behaviours. The direct and indirect effects of these shifts in social behaviour then propagated throughout the ecological network in different ways to transform the structure and dynamics of whole communities. Bacterial social traits are often encoded on mobile genetic elements and so may be rapidly lost or acquired by a bacterial population depending on environmental conditions^31^,^32^,^33^,^34^. Developing our understanding of the effects of bacterial niche construction and trait plasticity on community composition may, therefore, prove critical in the development of treatments aimed at manipulating our microbiota.

There has been a credibility gap in the scientific community with respect to microcosm experiments^35^, with many arguing that results obtained at such small scales lose relevance when extrapolated up to larger natural systems^36^. However, this criticism has eroded in the face of impressive results derived from microcosm experiments with significant ecological and evolutionary relevance^35^,^37^,^38^. Microcosm communities, particularly those from aquatic and soil ecosystems, have been found to reflect the dynamics of what we find in nature in response to environmental pressures^35^,^39^,^40^,^41^,^42^,^43^. One of the most fundamental aspects of a community is its trophic structure. The use of microcosms such as those used in our study, enabling complex multispecies interactions both within and among trophic levels, allow for a much more accurate representation of natural systems and the communities contained therein.

Research into their social behaviours has shown that bacteria are master manipulators of their surroundings, using collective behaviours to forge environments favouring their survival^12^,^14^. Loss of these social behaviours has been documented under numerous conditions^12^,^44^,^45^,^46^, but with little understanding of the consequences of this in a community context. Our results show that changes in these traits can affect the composition of the communities in which bacteria live as much as the canonical large community perturbation—species extinction. Much work to date has focused on the likelihood of extinctions following environmental change and their potential knock-on effects on communities^2^,^3^,^20^,^23^,^25^,^47^. However, changes in environmental conditions also have the potential to cause microevolution in microbial populations. Our findings suggest that microevolution may be an important, but currently overlooked, factor driving the community-level consequences of environmental change. Moreover, they also demonstrate clearly the potential for intraspecific genetic variability to be a key determinant of community dynamics. We conclude that integration of social trait plasticity into community ecology is critical to understand the structure, functioning and dynamics of microbial communities, with important implications for larger-scale systems.

## Methods

### Experimental design

Our experiment comprised six treatments, each consisting of 10 independent replicate microcosm communities: (1) a control treatment with all species present and containing *B. subtilis* WT; (2) removal of the apex predator *Didinium*; (3) removal of *B. subtilis*; (4) all species present with replacement of *B. subtilis* WT with the Δ*sinI* mutant, reducing biofilm production; (5) all species present with replacement of *B. subtilis* WT with the Δ*sinR* mutant, increasing biofilm production; (6) all species present with replacement of *B. subtilis* WT with the Δ*phoA* mutant, as a procedural control.

### Community assembly

**Figure 1:**
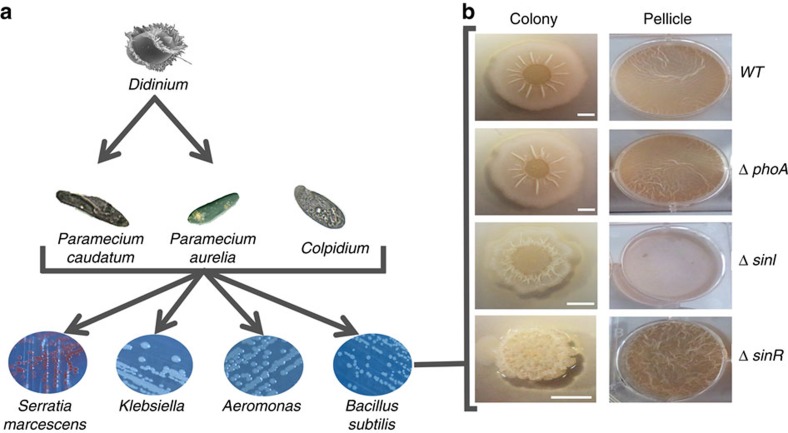
The composition of our experimental microcosms. (**a**) Trophic structure. Arrows indicate trophic relationships leading from consumer to prey organisms. (**b**) Effects of the Δ*sinI,* Δ*sinR* and Δ*phoA* mutations on biofilm formation and colony surface architecture in *B. subtilis*. Pellicle column depicts top-down images of microtitre wells in which cells have been grown in MSgg medium for 3 days at 22 °C. The colony column shows top-down images of Petri plates containing a 1.5%-Agar MSgg plate that was initially incubated overnight at 37 °C and later kept at room temperature. White scale bar, 4.6mm. Image credits: Dr Aaron J. Bell (*Didnium*), Yana Eglit (*P. aurelia*).

**Figure 2:**
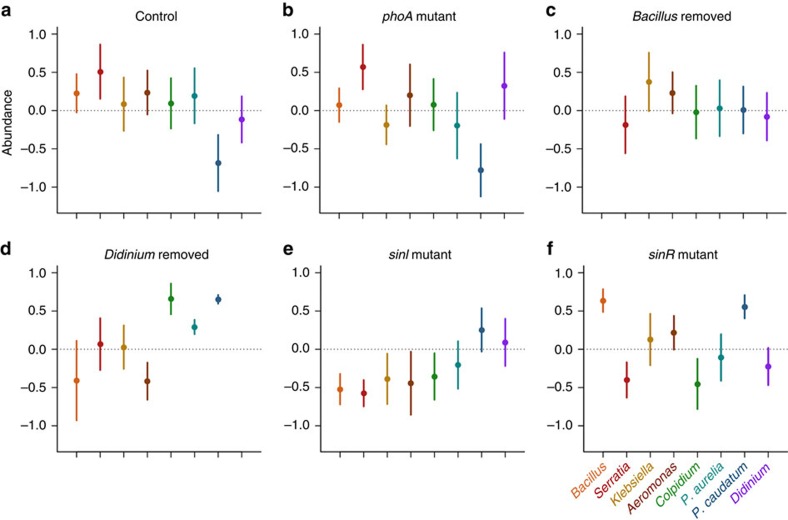
Community structure in our experimental treatments. Normalized (mean-standardized; overall mean represented by dotted line) abundances (mean±s.e.m, *n*=10) of each of *B. subtilis, S. marcescens, Klebsiella, Aeromonas, Colpidium, P. aurelia, P. caudatum* and *Didinium* in each experimental treatment (**a**–**f**).

**Figure 3:**
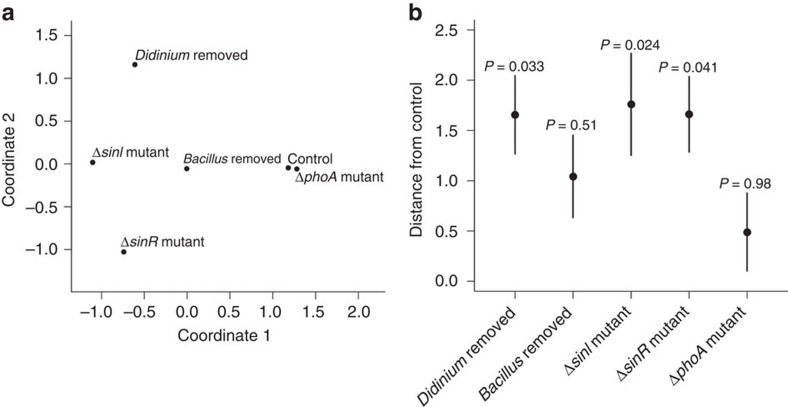
The extent of change in community structure caused by our experimental manipulations. (**a**) Non-metric multidimensional scaling ordination showing the variation in community structure among experimental treatments. Only the centroid of each experimental treatment is shown here for clarity. The ordination (stress=0.03) is based on a Euclidian distance matrix calculated from normalized (mean standardized) species abundances. (**b**) Euclidean distances between the centroid of each treatment and the control treatment (±bootstrapped s.e.m. from 10^4^ samples) and results of associated permutation tests (10^4^ permutations). *Didinium* and *B. subtilis* were excluded from these analyses as they are absent from some treatments as part of the experimental design.

**Figure 4:**
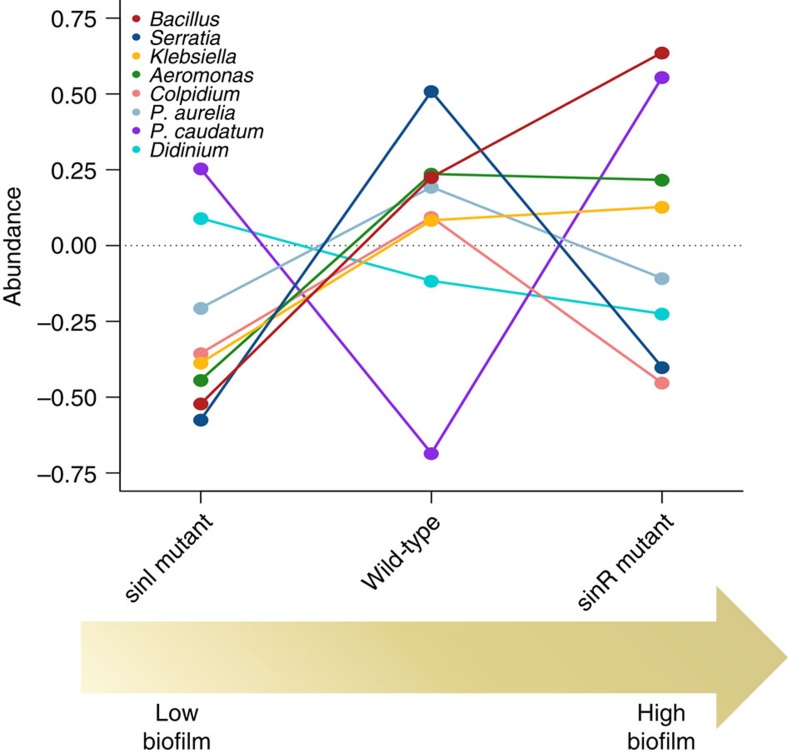
Shifts in species abundances along the observed biofilm production gradient in *B. subtilis*. Normalized (mean standardized) abundances of each of *B. subtilis, S. marcescens, Klebsiella, Aeromonas, Colpidium, P. aurelia, P. caudatum* and *Didinium* along an axis of sociality as regards biofilm production in *B. subtilis* from low to high.

Culture methods followed closely those of in refs 48, 49. Microcosms consisted of loosely capped 200-ml glass bottles with 15-g glass microbeads providing habitat structure. Each microcosm received 100 ml medium consisting of one protist pellet (Carolina Biological Supply, Burlington, NC, USA) per 1-l spring water and two wheat seeds to provide a slow-release nutrient source. All media were sterilized before use. Microcosms were maintained at 22 °C and under a 12:12-h light:dark cycle. Nutrients in the microcosms were replenished with weekly replacement of 7 ml of the microcosm volume with sterile medium and one additional sterile wheat seed. *Paramecium* and *Colpidium* species, generalist consumers with similar interaction strengths with each of the inoculated bacteria species (Fig. 1 and Supplementary Fig. 1; (refs 50, 51, 52, 53, 54, 55, 56, 57); MANOVA, Pillai's Trace=0.05, *F*_2,8_=2.5, *P =*0.14 (see *Primary consumer experiment* subsection below)), were obtained from Carolina Biological Supply (Burlington, NC, USA). *Didinium* were obtained from Sciento (Manchester, UK). Sources and strains of all bacterial strains used are listed in Supplementary Table 2.

Overnight cultures of strains NCIB3610 (WT), LSB369 (*sinI)*, LSB370 (*sinR*), LSB377 (*phoA*) and *S. marcescens* ATCC 29632 grown in TY medium (Luria–Bertani (LB) broth supplemented with 10 mM MgSO_4_ and 100 μM MnSO_4_ after autoclaving^58^ plus 100 μg ml^−1^ spectinomycin when appropriate), were diluted into fresh TY medium at OD_600_ (optical density) ∼0.03 and grown at 37 °C until late exponential phase (OD_600_∼1.0), at which time 1 ml of each bacterial culture was inoculated into 100-ml microcosm medium, according to the experimental set-up. *B. megaterium* (ATCC 19213) was also added but failed to establish in any microcosms.

Microcosms were then left for 48 h at 37 °C to facilitate growth of the bacteria and ensure sufficient numbers before the addition of the bacterivorous protists. Microcosms were inoculated with six pipette drops of stock cultures of protozoans: *P. caudatum*, *P. aurelia* and *Colpidium* (∼50–70 individuals of each species) and allowed to settle for 1 week at 22 °C. Predators (*Didinium*) were added (∼10 individuals) to appropriate microcosms 7 days after the addition of the bacterivores. The consumer protist cultures used for this experiment were laboratory cultures and therefore, while not inoculated with any bacterial populations, not entirely sterile; therefore, cultures were mixed thoroughly before addition of consumers to ensure that any bacteria present in the media had an equal chance of colonizing each microcosm. Both *Klebsiella* and *Aeromonas* colonized all microcosm units in this manner.

The point of addition of the predators is considered as Day 0 of the experiment with measurements taking place on Day 14. Samples were also taken on Day 7 to check for persistence of species and the presence of contaminants. As bacterial sampling is destructive, requiring vortexing of microcosms to strip biofilm and reduce within-mesocosm variability, only data from Day 14 are used in analyses. Pilot experiments, using the mutant- and predator-removed treatments, found that this period was sufficient for all organisms in the community to reach equilibrium densities, and a consistent difference between the treatments and control show that the effect was not transient (Supplementary Fig. 2). Protists were sampled by gently swirling the microcosms to homogenize contents and to suspend the protists, and up to 1-ml sample was examined using stereo (Olympus SZX9) and compound (Olympus BX60) microscopes. Rare species were counted in the entire sample and more numerous species were counted in appropriately diluted subsamples. Bacterial density was measured through direct colony counts on plates (of nutrient agar for all species and LB supplemented with spectinomycin for easy quantification of *sinI, sinR* and *phoA* strains) from appropriately diluted samples.

### Bacterial mutant strain construction

Chromosomal deletions were first created in the 168 background. Strains LSB362, LSB363 and LSB368 were generated using an adaptation of long flanking homology PCR. The protocol is modified from the published procedure^58^. In brief, the *spc* gene (encoding for spectinomycin resistance) was amplified from plasmid template pDG1726 (ref. 59) using primers spc fwd/spc rev (Supplementary Table 3). Two primer pairs were designed to amplify ∼750-bp DNA fragments flanking the region to be deleted at its 5′ and 3′ ends using Phusion Polymerase. These fragments contained ∼25-bp homologous to the *spc* cassette. Primer pairs sinI up fwd/sinI up rev (spc) and sinI do fwd (spc)/sinI do rev were used for strain LSB362; sinI up fwd/sinR up rev (spc) and sinR do fwd (spc)/sinR do rev were used for strain LSB363; phoA up fwd/phoA up rev (spc) and phoA do fwd (spc)/phoA do rev were used for strain LSB368. Overall, 150–200 ng of the flanking fragments and 250–300 ng of the resistance cassette were joined together using the Expand Long Template PCR System (Roche) and the specific up fwd and down rev primers. The resulting PCR product was used to transform *B. subtilis* 168. Transformants were screened by direct colony PCR, using the up-forward primer with a reverse primer annealing inside the *spc* resistance cassette.

Mutations in the NCIB3610 background (strains LSB369, LSB370 and LSB377) were created by SPP1-mediated transduction from strains LSB362, LSB363 and LSB368, respectively, as described previously^60^. Transductants were screened by direct colony PCR, using the up-forward primer with a reverse primer annealing inside the *spc* resistance cassette. The resulting strains (LSB369, LSB370 and LSB377) were verified using diagnostic PCR and DNA sequencing.

### Pellicle (biofilm)/colony morphology assays

Strains 168, NCIB3610, LSB369 (Δ*sinI::spc*), LSB370 (Δ*sinR::spc*) and LSB377 (Δ*phoA::spc*) were cultivated in LB medium^61^ until mid-exponential growth (OD_600_∼0.5) at which time 10 μl of each culture was inoculated into 10 ml of MSgg medium^62^ in six-well plates, which were then incubated at room temperature (pellicle assay), or 5 μl was spotted on a 1.5% Agar MSgg plate that was initially incubated overnight at 37 ^o^C and later kept at room temperature (colony morphology).

### Primary consumer experiment

We established a subsidiary experiment to test whether the individual effects of the three primary consumer species used in our experiment on bacterial community structure varied either among each other or among communities containing the different *B. subtilis* phenotypes (WT, Δ*sinI* and Δ*sinR*). The experiments were performed in 64-well plates in 2-ml cells. Each cell contained sterile protist medium (identical to that used in the microcosms) and 100 μl of *Klebsiella*, *Serratia* and *Aeromonas* strains. To this was added 100 μl of one of the three *B. subtilis* phenotypes used in the experiment. Plates were incubated for 24 h, after which time five individuals of either *P. caudatum, P. aurelia* or *Colpidium* were added to cells. Each treatment was replicated three times. Control plates with no consumers were also included for comparison and to enable quantification of interaction strengths between each primary consumer species and their bacterial prey. After 2 days, the bacteria were enumerated on nutrient agar from appropriately diluted subsamples.

### Statistical analyses

We used MANOVA to test whether our treatments significantly affected the structure of the microcosm communities. All abundances were log-transformed and mean-standardized (expressed in units of s.d.) before analysis. To assess the extent of change in community composition among treatments, we used a permutation test (with 10^4^ permutations) to test for differences in the location of community centroids between each treatment and the control. We then examined differences in the relative magnitudes of these changes in community composition by bootstrapping, using 10^4^ samples of the data taken with replacement. We tested for correlations in species abundances with the axis of biofilm formation phenotype using Spearman's rank correlation tests. Total interaction strengths between each bacterial species and each of the three primary consumers used in our experiment were quantified as the natural logarithm of the ratio of untransformed densities of each bacterial species in each microcosm containing each primary consumer species in isolation to their mean untransformed density in control microcosms without primary consumers^63^.

## Additional information

**How to cite this article:** McClean, D. *et al.* Single gene locus changes perturb complex microbial communities as much as apex predator loss. *Nat. Commun.* 6:8235 doi: 10.1038/ncomms9235 (2015).

## Supplementary Material

Supplementary Information Supplementary Figures 1-2 and Supplementary Tables 1-3
