# Supplementary material for: Single gene locus changes perturb complex microbial communities as much as apex predator loss
Source: Nat Commun. 2015 Sep 10;6:8235. doi: 10.1038/ncomms9235 (PMC4579780; doi:10.1038/ncomms9235)
Supplement: Supplementary Information — Supplementary Figures 1-2 and Supplementary Tables 1-3 [file ncomms9235-s1.pdf]

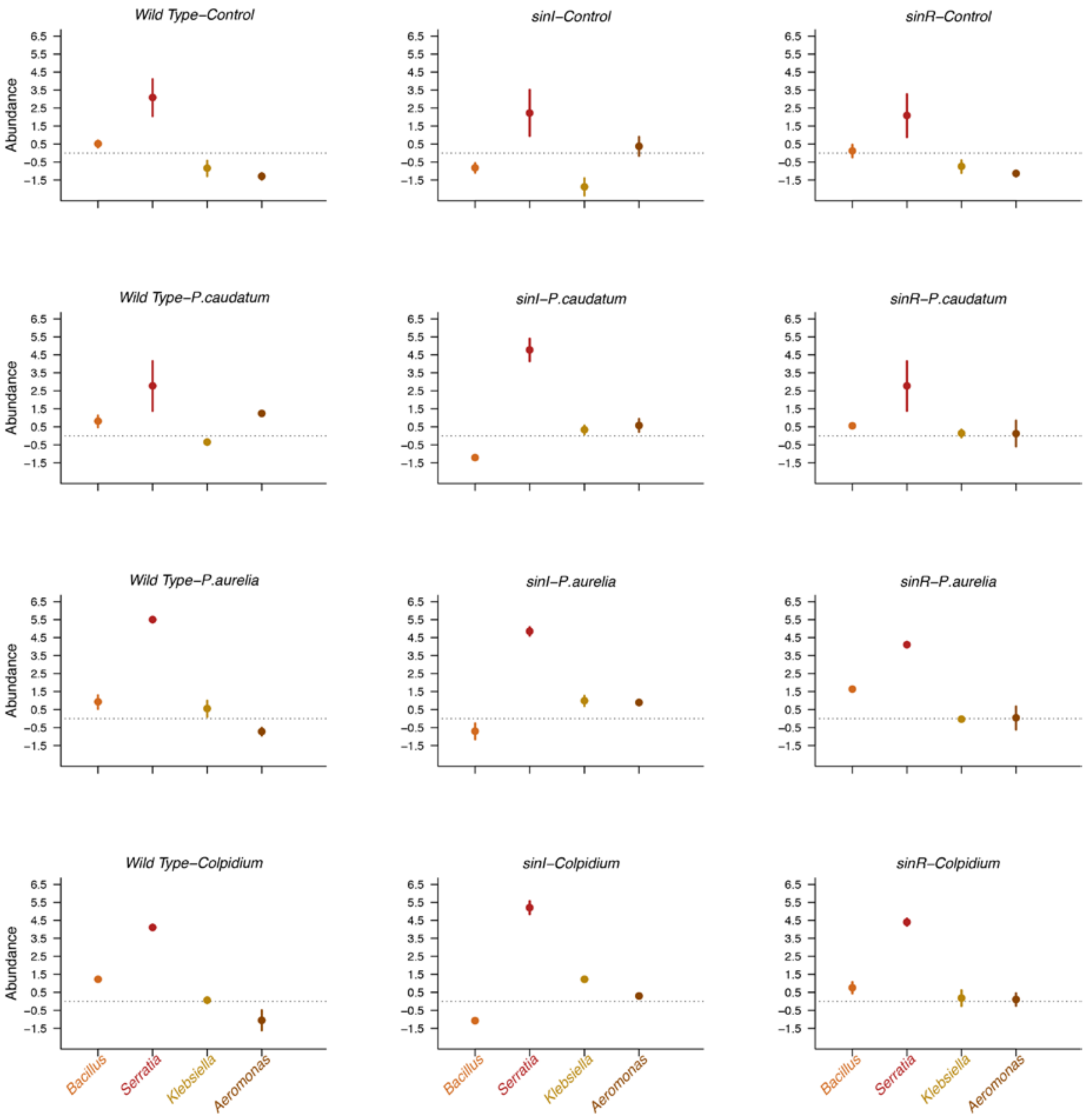

Supplementary Figure 1. Individual effects of each primary consumer species on bacterial community structure. Normalised (mean standardised) abundances (mean ± s.e.m, *n* = 3) of each bacterial species (*Bacillus*, *Serratia*, *Klebsiella* and *Aeromonas*) in microcosms containing no consumers (‘Control’) or one of the three primary consumer species used in the experiment (*P.caudatum* [second row from top], *P. Aurelia* [third row from top], *Colpidium* [bottom row]) across each of the three *Bacillus* treatments (wild type [left column], *sinI* [middle column], *sinR* [right column]).

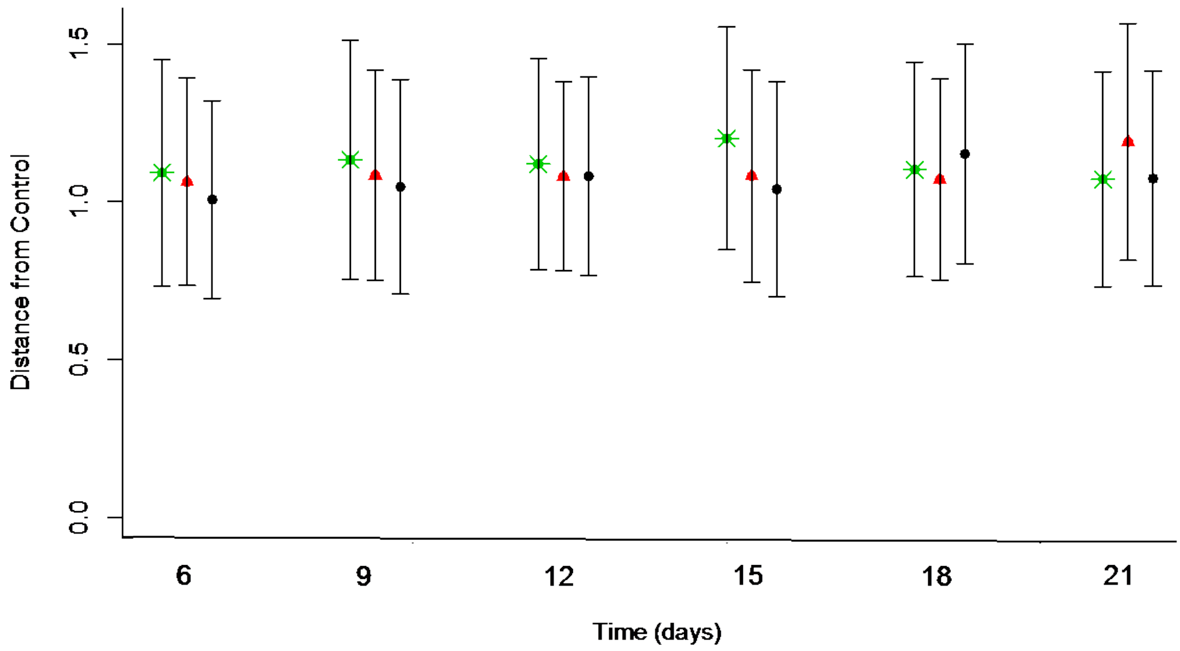

Supplementary Figure 2. Euclidean distances between the centroid of the control treatment and each of the Δ*sinI* (black circles), Δ*sinR* (red triangles) and *Didinium* removed (green stars) treatments (± bootstrapped sd. from 10^4 samples) across six time points (over 21 days) in pilot experiments.

Supplementary Table 1. Results of Spearman rank correlation tests for normalised densities of each species along a gradient of biofilm formation (see Fig. 4 in main text).

**Species**	**rho**	***P***
B. subtilis	0.604	< 0.001
S. marcescens	0.076	0.69
Klebsiella	0.191	0.31
Aeromonas	0.013	0.95
P. aurelia	0.113	0.55
P. caudatum	0.125	0.51
Colpidium	0.259	0.17
Didinium	-0.165	0.38

Supplementary Table 2. Bacterial strains used in the study.

Strain	Description	Source or reference
168 (*B.subtilis*)	*trpC2*	Laboratory stock
NCIB3610 (*B.subtilis*)	Wild-type isolate	BGSC 3A1
Serratia marcescens	Wild-type isolate	ATCC® 29632™
LSB362 (*B.subtilis*)	*trpC2* Δ*sinI*::*spc*	LFH →168
LSB363 (*B.subtilis*)	*trpC2* Δ*sinR*::*spc*	LFH →168
LSB368 (*B.subtilis*)	*trpC2* Δ*phoA*::*spc*	LFH →168
LSB369 (*B.subtilis*)	NCIB3610 Δ*sinI*::*spc*	SPP1 transduction →NCIB3610
LSB370 (*B.subtilis*)	NCIB3610 Δ*sinR*::*spc*	SPP1 transduction →NCIB3610
LSB377 (*B.subtilis*)	NCIB3610 Δ*phoA::spc*	SPP1 transduction →NCIB3610

Supplementary Table 3. Primers used in the study.

**Primer #**	**Primer name**	**Primer sequence**
oLS025	spc fwd	GACTGGCTCGCTAATAACGTAACGTGACTGGCAAGAG
oLS026	spc rev	CGTAGCGAGGGCAAGGGTTTATTGTTTTCTAAAATCTG
oLS295	phoA up fwd	GCCGTTAATCGATAAAGTTTGTCC
oLS298	phoA do rev	GGAAACCAAGATTATGAATGAGG
oLS643	sinI up fwd	AAACAAAATGGGAGATTTACCAG
oLS646	sinI do rev	GCAAGCGGTAAAGTCAATGTAGC
oLS654	sinR do rev	TTAGTTGGAGGAGGAACATGGG
oLS785	sinI up rev (spc)	CGTTACGTTATTAGCGAGCCAGTCTTCTTCATGCAGTTTCTCCTCC
oLS786	sinI do fwd (spc)	CAATAAACCCTTGCCCTCGCTACGCCGTAAATCCTTTCTGAATGTGC
oLS787	sinR up rev (spc)	CGTTACGTTATTAGCGAGCCAGTCTGGCCAATCAATGTCATCACC
oLS788	sinR do fwd (spc)	CAATAAACCCTTGCCCTCGCTACGAGTAGTGCCTGAGCAGAGGC
oLS801	phoA up rev (spc)	CGTTACGTTATTAGCGAGCCAGTCAAGGACAGAAACAGCGGCGATTGG
oLS802	phoA do fwd (spc)	CAATAAACCCTTGCCCTCGCTACGGATTGATTAACAATACGGACCAGGC
